# The Enterics for Global Health (EFGH) *Shigella* Surveillance Study in Kenya

**DOI:** 10.1093/ofid/ofad654

**Published:** 2024-03-25

**Authors:** Richard Omore, Alex O Awuor, Billy Ogwel, Caleb Okonji, Catherine Sonye, Caren Oreso, Victor Akelo, Manase Amollo, Isaiah Ogudi, Raphael O Anyango, Marjorie Audi, Evans Apondi, Laura Riziki, Lilian Ambila, Nasrin Dilruba, Erick Muok, Stephen Munga, John B Ochieng, Karen L Kotloff

**Affiliations:** Centre for Global Health Research, Kenya Medical Research Institute, Kisumu, Kenya; Centre for Global Health Research, Kenya Medical Research Institute, Kisumu, Kenya; Centre for Global Health Research, Kenya Medical Research Institute, Kisumu, Kenya; Centre for Global Health Research, Kenya Medical Research Institute, Kisumu, Kenya; Centre for Global Health Research, Kenya Medical Research Institute, Kisumu, Kenya; Centre for Global Health Research, Kenya Medical Research Institute, Kisumu, Kenya; Department of Clinical Medicine, Liverpool School of Tropical Medicine, Kisumu, Kenya; Centre for Global Health Research, Kenya Medical Research Institute, Kisumu, Kenya; Centre for Global Health Research, Kenya Medical Research Institute, Kisumu, Kenya; Centre for Global Health Research, Kenya Medical Research Institute, Kisumu, Kenya; Centre for Global Health Research, Kenya Medical Research Institute, Kisumu, Kenya; Centre for Global Health Research, Kenya Medical Research Institute, Kisumu, Kenya; Centre for Global Health Research, Kenya Medical Research Institute, Kisumu, Kenya; Centre for Global Health Research, Kenya Medical Research Institute, Kisumu, Kenya; Center for Vaccine Development and Global Health, University of Maryland School of Medicine, Baltimore, Maryland, USA; Centre for Global Health Research, Kenya Medical Research Institute, Kisumu, Kenya; Centre for Global Health Research, Kenya Medical Research Institute, Kisumu, Kenya; Centre for Global Health Research, Kenya Medical Research Institute, Kisumu, Kenya; Center for Vaccine Development and Global Health, University of Maryland School of Medicine, Baltimore, Maryland, USA

## Abstract

**Background:**

Although *Shigella* is an important cause of diarrhea in Kenyan children, robust research platforms capable of conducting incidence-based *Shigella* estimates and eventual *Shigella-*targeted clinical trials are needed to improve *Shigella*-related outcomes in children. Here, we describe characteristics of a disease surveillance platform whose goal is to support incidence and consequences of *Shigella* diarrhea as part of multicounty surveillance aimed at preparing sites and assembling expertise for future *Shigella* vaccine trials.

**Methods:**

We mobilized our preexisting expertise in shigellosis, vaccinology, and diarrheal disease epidemiology, which we combined with our experience conducting population-based sampling, clinical trials with high (97%–98%) retention rates, and healthcare utilization surveys. We leveraged our established demographic surveillance system (DSS), our network of healthcare centers serving the DSS, and our laboratory facilities with staff experienced in performing microbiologic and molecular diagnostics to identify enteric infections. We joined these resources with an international network of sites with similar capabilities and infrastructure to form a cohesive scientific network, designated Enterics for Global Health (EFGH), with the aim of expanding and updating our knowledge of the epidemiology and adverse consequences of shigellosis and enriching local research and career development priorities.

**Conclusions:**

*Shigella* surveillance data from this platform could help inform *Shigella* vaccine trials.

## Teaser

A diarrhea-specific research platform able to perform population-based enumeration and hospital-based surveillance plus support mentorship of young scientists has been established in western Kenya to support Enterics for Global Health–*Shigella* surveillance and future vaccine trials.

*Shigella* is among the most common causes of diarrhea morbidity among children aged <5 years in Kenya [1]. Furthermore, with the use of more sensitive molecular methods, the relative contribution of *Shigella* has even increased 2- to 4-fold compared to culture in Kenya [1–3]. Kenya is among the lower-middle-income countries (LMICs) where *Shigella*-resistant isolates to first-line antibiotics and high rates of inappropriate antibiotic prescription are common [4–6]. *Shigella* vaccines have the potential to reduce *Shigella* transmission as well as reduce diarrhea and dysentery – the two most common reasons for antibiotic use. Several *Shigella* vaccines are in the pipeline [7] to reduce morbidity and mortality associated with *Shigella* diarrhea, especially in high-disease-burden settings such as Kenya.

Investing in and prioritizing specific interventions such as vaccines in Kenya requires precise, country-specific data on disease burden and consequences to support policy makers on making decisions on cost against benefit. Such data may contribute toward the achievement of the United Nations universal Sustainable Development Goal 3—good health and well-being as a priority both locally and internationally. Such data could also help inform public health interventions, vaccine trials, and impact evaluation including but not limited to decisions on antibiotic use for enteric bacterial pathogens. Investing in research platforms with demonstrated ability to support vaccine trials as well as pre- and post-vaccine disease burden estimates while providing opportunity for scientific mentorship for young scientists is a high priority in Kenya. This article aims to describe the composition and features of the Kenyan site of the Enterics for Global Health (EFGH) *Shigella* surveillance study, a protocol that was reviewed and approved by the Kenya Medical Research Institute (KEMRI) Scientific and Ethical Review Unit (SERU#4362) and the Institutional Review Board of the University of Maryland, Baltimore, Maryland, USA (HP-000982), established with a goal to support future *Shigella* vaccine trials.

## SITE LOCATION AND DEMOGRAPHIC DESCRIPTION

**Figure 1.**
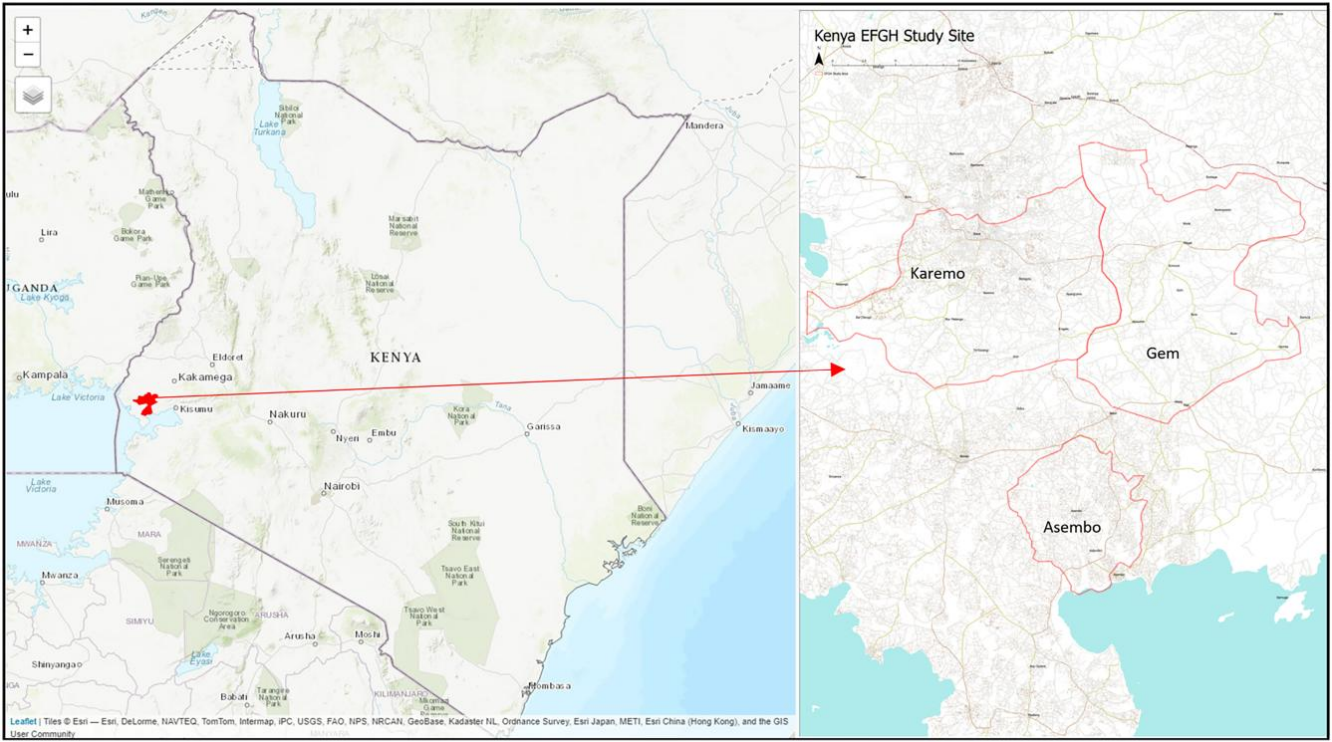
Map of Enterics for Global Health (EFGH)–*Shigella* Kenya study area including diarrhea case surveillance sentinel facilities, western Kenya, 2022.

The EFGH-*Shigella* site in Kenya is located within Gem, Karemo, and Asembo communities in rural western Kenya. Although the EFGH study site overlaps with the Health and Demographic Surveillance System (HDSS) areas operated by KEMRI [8], it is restricted within specific villages that also form clusters, unlike HDSS, which covers a much wider area as described elsewhere [8]. The Kenyan EFGH-*Shigella* disease surveillance site lies northeast of Lake Victoria and is approximately 40 km from the KEMRI Centre for Global Health Research (CGHR) campus in Kisumu where the central offices and laboratories for the research platform are situated (Figure 1). The last Kenya population and housing census was conducted in 2019 and enumerated 47 564 296 people with an estimated intercensal growth rate of 2.2% [9]. The study area has 2 rainy seasons, in April–May and November–December, and an annual rainfall of approximately 2155 mm. Kenya reported a per capita gross domestic product of US$2081.80 in 2021; the main economic activity is subsistence farming, performed in 60% of households [8, 10]. Most of the houses in the study area are made of mud, cement, or brick with roofs of iron sheets or thatched grass [8]. Most households use unimproved water sources (51.5%), even though 65.4% of households claim to treat their drinking water. In general, 77.4% of households use a traditional pit latrine as the main waste disposal means. The level of education among residents was 58.5% and 30.3% for primary and secondary education, respectively (unpublished HDSS data). From the recent Kenya Demographic and Health Survey, 18% of children aged <5 years were stunted and 5% were wasted nationally. However, in Siaya County where the EFGH study will take place, 19.2% of children were stunted, 1.7% were wasted, and 7.0% were underweight [11].

The routine immunization schedule in Kenya for childhood vaccines is shown in Table 1 [12]. The latest additions to the National Vaccines and Immunization Program include the Rotarix rotavirus vaccine and the RTS,S/AS01 malaria vaccine, which were introduced in July 2014 and September 2019, respectively. Although Rotarix vaccine is administered in 2 doses at age 6 weeks and 10 weeks, Kenya recently transitioned from Gavi support and opted to switch to a cheaper Rotavac vaccine option, which is administered at 6, 10, and 14 weeks [13]. Malaria vaccine is administered in 4 doses (6, 7, 9, and 24 months). Some of the lessons learned from the rotavirus vaccine introduction include the need for creating awareness to the community and ensuring sustainability by addressing global supply challenges and cost-saving for countries that graduated or are in transition from Gavi support [12, 14].

**Table 1.** Routine Immunization Schedule in Kenya, Adopted From Kenya National Immunization Policy Guidelines 2023

Contact	Age of Child	Vaccine (Dose Number)	Dosage	Route
1	At birth or at first contact	BCG	0.05 mL 0.1 mL >1 y	Intradermal
	At birth or at first contact (within the first 2 wk of life)	OPV birth dose (bivalent)	2 drops	Oral
2	At 6 wk of life or at first contact after 6 wk	OPV (1)	2 drops	Oral
		DPT-HepB-Hib (1)	0.5 mL	IM into the upper outer aspect of left thigh
		PCV10 (1)	0.5 mL	IM into the upper outer aspect of right thigh
		Rotavirus (1)	0.5 mL (5 drops)	Oral
3	At 10 wk or 4 wk after OPV 1st dose, DPT-HepB-Hib 1st dose, and PCV10 1st dose	OPV (2)	2 drops	Oral
		DPT-HepB-Hib (2)	0.5 mL	IM into the upper outer aspect of left thigh
		PCV10 (2)	0.5 mL	IM into the upper outer aspect of right thigh
		Rotavirus (2)	0.5 mL (5 drops)	Oral
4	At 14 wk or 4 wk after OPV 2nd dose, DPT-HepB-Hib 2nd dose, and PCV10 2nd dose	OPV (3)	2 drops	Oral
		DPT-HepB-Hib (3)	0.5 mL	IM into the upper outer aspect of left thigh
		PCV10 (3)	0.5 mL	IM into the upper outer aspect of right thigh
		IPV	0.5 mL	IM into the upper outer aspect of right thigh, 2.5 cm from PCV10 dose 3 site
		Rotavirus (3)	0.5 mL (5 drops)	Oral
5	At 6 mo	Vitamin A	100 000 IU	Oral
		Measles-rubella	0.5 mL	SC right upper arm (deltoid muscle) in the event of measles-rubella outbreak or HIV-infected infants who are not severely immunosuppressed
		RTS,S/AS01 malaria vaccine (1) (high-risk counties)	0.5 mL	IM left deltoid muscle
6	At 7 mo	RTS,S/AS01 malaria vaccine (2) (high-risk counties)	0.5 mL	IM left deltoid muscle
7	At 9 mo or first contact after 9 mo	Measles-rubella (1)	0.5 mL	SC into the right upper arm (deltoid muscle)
		Yellow fever (high-risk counties)	0.5 mL	SC into the left upper arm (deltoid muscle)
		RTS,S/AS01 malaria vaccine (3) (high-risk counties)	0.5 mL	IM left deltoid muscle
8	At 12 mo of age	Vitamin A	200 000 IU	Oral
9	At 18 mo or first contact after 18 mo	Measles-rubella (2)	0.5 mL	SC into the right upper arm (deltoid muscle)
	At 18 mo	Vitamin A 200 000 IU	One capsule	Oral
10	At 24 mo	RTS,S/AS01 malaria vaccine (4) (high-risk counties)	0.5 mL	IM left deltoid muscle
11	At 10 y for girls (extend to 14 y for catch-up)	HPV (1)	0.5 mL	IM left deltoid muscle
12	10 y, 6 mo (or 6 mo after HPV 1st dose)	HPV (2)	0.5 mL	IM left deltoid muscle

Source: Kenya Ministry of Health [12].

Abbreviations: DPT, diphtheria-pertussis-tetanus; HepB, hepatitis B; Hib, *Haemophilus influenzae* type b; HIV, human immunodeficiency virus; HPV, human papillomavirus; IM, intramuscular; IPV, inactivated polio vaccine; OPV, oral polio vaccine; PCV10, 10-valent pneumococcal conjugate vaccine; SC, subcutaneous.

The Kenyan healthcare sector relies on funding from the following sources: public sector (government), private sector, patient payments and donors (faith-based organizations and nongovernmental organizations), and health insurance schemes. The Kenya government has also initiated a piloted universal health coverage (UHC) in select counties including Kisumu County, which strives for quality and effective health provision for all at minimal financial cost [15]. One of the most critical challenges faced by the healthcare system is to generate efficient, fair, and sustainable financing mechanisms that guarantee UHC to all.

## CATCHMENT AREA MAP AND OVERVIEW OF RECRUITMENT FACILITIES

**Figure 2.**
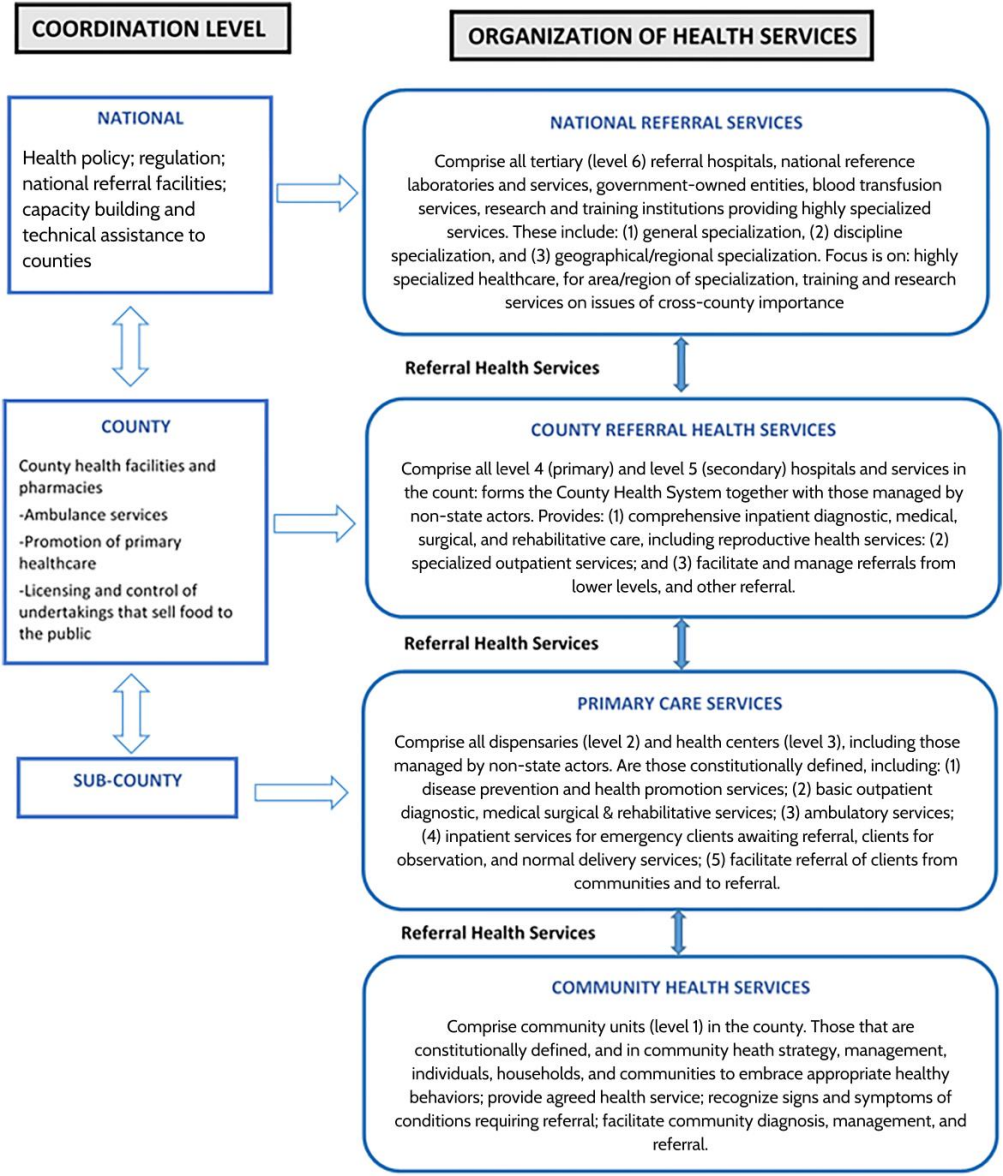
Organization of health service delivery and referral system in Kenya (adopted from the Kenya Health Policy 2014–2030 [17]).

Kenya operates a 6-level health system composed of several health facilities including community services (level 1), dispensaries and clinics (level 2), health centers and maternity and nursing homes (level 3), subcounty hospitals and medium-sized private hospitals (level 4), county referral hospitals and large private hospitals (level 5), and national referral hospitals and large private teaching hospitals (level 6). Primary healthcare services are primarily provided at levels 1–3. The key healthcare service providers are the Ministry of Health (MoH) and parastatal organizations, as well as the private sector, which comprises private for-profit, nongovernmental organizations, and faith-based organization facilities [16]. The organization of the health service delivery and referral system in Kenya is shown in Figure 2.

Six recruitment healthcare facilities in the EFGH Kenya study catchment area were selected based on the top preference of caretakers for seeking care for their child's diarrheal illnesses and being the highest enrolling of the 15 facilities from the Vaccine Impact on Diarrhea in Africa (VIDA) study [18]. Specifically, Siaya County Referral Hospital is a level 4 hospital, located in the northern part of the county; it offers emergency, diagnostic, inpatient, and outpatient services and is the main referral facility in Siaya County. Ongielo, Akala, and Dienya are level 3 government-operated health facilities offering both inpatient and outpatient services. Bar Agulu is a level 2 government health dispensary facility offering outpatient services only. Lwak Mission Hospital (LMH) is a nongovernmental private facility operated by the Catholic Mission. It provides both inpatient and outpatient medical services and is the least usually affected by drug shortages since it does not depend on the government drug supply chain except for routine distribution of vaccines as part of the national immunization program (Figure 1). The EFGH staff have been integrated into all these facilities within the maternal and child health/outpatient clinics where they work strategically to attend to all children aged <5 years who seek care from those units, mainly from the EFGH catchment population and neighborhoods. The EFGH Consortium offers buffer drug supplies (oral rehydration solution [ORS], zinc, intravenous [IV] fluids, antibiotics recommended for diarrhea management, and antimalarial drugs) to complement the government supplies in times of drug shortages to all children regardless of study participants. As part of the study ethical obligation, laboratory results that include culture and antimicrobial susceptibility results for *Shigella* isolates are communicated back to the enrolling clinician to help guide further clinical management of the patients. Furthermore, where applicable, the MoH department of outbreak investigation is equally notified for further action.

## DIARRHEA MANAGEMENT

The management of childhood diarrhea in Kenya is guided by the Kenya Basic Pediatric Protocol 2016, which largely conforms with the World Health Organization (WHO) Integrated Management of Childhood Illnesses (IMCI) guideline [19, 20]. The implementation of these guidelines are reinforced by the MoH guidelines, as they are recommended for all healthcare providers in the country. The mainstay of treatment of diarrhea in children is ORS, zinc, and IV fluid in severe dehydration. The guidelines have plan A, B, and C depending on the degree of the dehydration (severe, some, and no dehydration) as described in the IMCI guideline [20]. The etiology of the diarrhea also plays a role in management of the child, with antibiotics (ciprofloxacin, azithromycin, and ceftriaxone) recommended for shigellosis or cholera, consistent with WHO guidelines [20]. Management of children with severe dehydration using plan C includes starting with IV fluid immediately (preferably Ringer's lactate solution [100 mL/kg]) divided as per guideline, reassessed every 15–30 minutes, and giving ORS (5 mL/kg/hour) as soon as the child can drink, but reclassifying dehydration after 6 hours of rehydration for infants. In plan B, those children with some dehydration, management entails administering recommended ORS in the clinic over 4 hours then reclassifying dehydration after 4 hours and continuing with plan A, B, or C. Last, plan A covers the management of children classified as having diarrhea with no dehydration, in which the caretakers are advised to increase food and fluid intake to prevent dehydration and set a return date for follow-up. Management of children with severe malnutrition and diarrhea with any dehydration is recommended to be done as inpatient and for children to be given nutritional supplements. Detailed management in the guideline is shown in Table 2 and further detailed in the above guidelines. Additionally, for all children with diarrhea with any dehydration, zinc supplementation should be administered for 10–14 days with the following dosage: 10 mg/kg body weight daily for age ≤6 months and 20 mg/kg body weight for age >6 months. Children with dysentery confirmed or blood in stool should be given ciprofloxacin (15 mg/kg) twice daily for 3 days or, based on local sensitivity, IV/intramuscular ceftriaxone at 50–80 mg/kg per day for 3 days (if child is severely ill or as second-line treatment).

**Table 2.** Kenya Site Diarrhea Case Management as per World Health Organization Integrated Management of Childhood Illnesses 2005 Guidelines as Adopted by the Kenya Basic Pediatric Protocol, Nairobi, Kenya 2016

Treatment	Country or Site-Specific Guideline	
**Dehydration management (without severe acute malnutrition)**		
Severe	Start IV fluid immediately (preferably Ringer’s lactate solution [100 mL/kg]) Reassess every 15–30 min Give ORS (5 mL/kg/h) as soon as the child can drink Reclassify dehydration after 6 h in infants and 3 h in children and continue with A, B, C plan	Age <12 mo: 30 m:/kg in 1 h followed by 70 mL/kg over 5 h (repeat if radial pulse if still very weak or not detectable) Age 12 mo to 5 y: 30 mL/kg in 30 min followed by 70 mL/kg over 2.5 h (repeat if the radial pulse is still very weak or not detectable)
Some	Give recommended ORS in clinic over 4 hours. Re-classify dehydration after 4 hours and continue with A, B, C plan	Age ≤ 4 mo: < 6 kg:200-400ml; 4-≤12 mo:6-<10 kg:400-700 ml; 12 mo-≤2 y:10-≤12 kg: 700-900 ml; 2 y-≤ 5 y: 12-19 kg: 900-1400 ml (Use child's age only when weight is not known. The appproximate amount of ORS required (in ml) can also be calculated by multiplying the child'd weight (in kg) by 75.)
None	Increase food and fluid intake to prevent dehydration	Advise when to return
**Dehydration management (with severe acute malnutrition and no shock)**		
Severe	Give ReSoMal (or half-strength standard low ORS with added potassium and glucose) 5 mL/kg every 30 min for the first 2 h Give ReSoMal 5–10 mL/kg/h for the next 4–10 h on alternate hours with F75 If rehydration still required at 10 h, give starter F75 instead of ReSoMal, at the same times	
Some	Give ReSoMal (or half-strength standard low ORS with added potassium and glucose) 5 mL/kg every 30 min for the first 2 h 5–10 mL/kg/h for the next 4–10 h on alternate hours with F75 If rehydration still required at 10 h, give starter F75 instead of ReSoMal, at the same times	
None	…	…
**Therapeutic zinc**		
Population: All children	Zinc supplementation for 10–14 d Age ≤6 mo: 10 mg/d Age >6 mo: 20 mg/d	
**Antibiotics**		
Population; Dysentery or *Shigella* upon culture confirmation	Ciprofloxacin (15 mg/kg) twice daily for 3 d or based on local sensitivity IV/IM ceftriaxone at 50–80 mg/kg/d for 3 d (if child is severely ill or as second-line treatment)	
Population: Suspected cholera (age ≥2 y + severe dehydration + cholera present in area)	Erythromycin (12 mg/kg) 4 times a day for 3 d Ciprofloxacin 10–20 mg/kg twice per day for 5 d Cotrimoxazole (4 mg/kg trimethoprim and 20 mg/kg sulfamethoxazole) twice a day In addition, manage with plan C for severe dehydration as stated above	

Sources: Kenya Ministry of Health [19], World Health Organization [20].

Abbreviations: F75, Formula-75; IM, intramuscular; IV, intravenous; ORS, oral rehydration solution; ReSoMal, rehydration solution for malnutrition.

Adherence to diarrhea guidelines is suboptimal in Kenya. In the recently conducted VIDA study occurring at the same 6 health facilities, the proportion of children with no, some, and severe dehydration who received recommended ORS was 77.1%, 86.7%, and 88.2% [21]. Additionally, the proportion who received recommended zinc was 96.4%, 95.9%, and 93.0%, respectively. Finally, the proportion of children with severe dehydration who received recommended IV rehydration was 14.3%. In 1 of our previous studies in the same setting, we observed that 34.2% of diarrheal visits to health facilities had an inappropriate antibiotic prescription [6]. More recently, in the VIDA study, Kenya had 25.1% of moderate to severe diarrhea (MSD) cases with no apparent antibiotic indicated being prescribed antibiotics; cotrimoxazole (29/70 [41.4%]) and metronidazole (19/70 [27.1%]) were the most frequently prescribed antibiotics [4]. Some challenges to adherence of IMCI guidelines in the management of childhood diarrhea in our setting include commodity availability (recommended IV fluids, drugs, IV cannulas, etc), inadequate diagnostic capacity, healthcare workers and caregiver factors, and less stringent enforcement of policy guidelines [22, 23]. These observations are not dissimilar to those commonly observed in other resource-poor settings located in other LMICs [24].

## HISTORICAL *SHIGELLA* INCIDENCE, PREVALENCE, AND ANTIMICROBIAL RESISTANCE DATA

In the Global Enteric Multicenter Study (GEMS), conducted between 2008 and 2012 in the same setting, *Shigella* spp. was isolated by culture from 7.3% (130/1778) of stool specimens collected from children aged 0–59 months presenting with MSD, of which *Shigella flexneri* and *Shigella sonnei* were the predominant serotypes, accounting for 61% and 18%, respectively, and such a pattern was consistent with observations from other GEMS sites [1, 25]. However, when a randomly selected subset (n = 912 [51%]) of the 1778 GEMS Kenya samples from MSD cases was reanalyzed by quantitative polymerase chain reaction (qPCR) using TaqMan Array Card (TAC), the isolation rate increased significantly. The *Shigella* results from TAC analysis were classified as highly diarrhea associated (positive for *Shigella* if cycle threshold [Ct] values were ≤27.9 and negative if Ct values were >27.9) and diarrhea associated (positive for *Shigella* if Ct values were ≤33.1 and negative if Ct values were >33.1). In total, 148 of the 912 (16.2%) samples tested by TAC were classified as highly diarrhea-associated *Shigella-*positive MSD cases. However, 238 of the 912 (26.1%) samples tested by TAC were classified as diarrhea associated with *Shigella*-positive MSD cases, suggesting that use of TAC increased the yield of *Shigella* >3-fold [2]. VIDA, a follow-up study after GEMS, documented that the proportion of MSD cases in which *Shigella* was detected at diarrhea-associated quantities by qPCR compared to culture was 21.8% versus 4.4% [18]; these observations are consistent with GEMS findings [2, 3], reaffirming the high sensitivity of qPCR in detecting *Shigella* from stool. The resistance patterns were largely similar between GEMS and VIDA, with antimicrobial resistance data from the VIDA study showing that resistance of *Shigella* isolates in decreasing order was 127 (97.7%), 76 (58.5%), 2 (1.5%), and 1 (0.8%) 0 for trimethoprim-sulfamethoxazole, ampicillin, nalidixic acid, and azithromycin, respectively. No resistance was observed for ceftriaxone [3]. While previous studies have shown diarrhea seasonality aligning with the rainfall pattern, climate change is likely an important driver influencing diarrhea epidemiology and even etiology-specific prevalence in Kenya, consistent with observations from other similar settings in Africa as described elsewhere [26–28].

## HISTORICAL HEALTHCARE-SEEKING PATTERNS FOR CHILDHOOD DIARRHEA

During GEMS, Kenya was the only site that conducted a whole population survey on the health utilization and attitude patterns in our HDSS area between 22 May 2009 and 31 December 2010. Overall care seeking outside home for MSD was 82.0% (2675/3632), while care seeking specifically from a healthcare facility was 61.9% (1657/2675) [29]. From the recently conducted Kenya Demographic and Health Survey of 2022, only 57% of caretakers sought care for their child's diarrheal illness at a healthcare facility [11]. Despite that care is being offered at no cost at LMH for participants enrolled in the US Centers for Disease Control and Prevention–funded population-based infectious disease surveillance, up to 42% of the study participants prefer seeking care from other health facilities where they have to pay for the same services instead [30]. Such observations suggest that free medical care may not be exclusively responsible for increase in healthcare seeking, a phenomenon that might have multifactorial drivers in this setting [31]. Other factors that might influence healthcare seeking could also include access to National Insurance Hospital Fund–accredited facilities, which cover part of treatment cost. Furthermore, from our site experience and unpublished data from GEMS and VIDA, several factors—including but not limited to (1) distance to the healthcare facilities, (2) patient management that includes quality and the type of services offered, and (3) attitude of the healthcare workers at the healthcare facility—could have substantial impact on healthcare seeking from the local healthcare facilities. Such observations are in part supported and align with findings from another morbidity surveillance from the same setting [32]. The Kenyan MoH through the community health strategy (CHS) encourages integrated community case management (iCCM) of mild cases of diarrhea. Using community health extension workers and community health volunteers, the MoH through CHS provides preventive and curative interventions at the community level using iCCM guidelines. Caretakers are encouraged to seek medical care at healthcare facilities if the child presents with severe disease including danger signs and, in the context of diarrhea, either dysentery or dehydration signs [33]. Comprehensive understanding of healthcare-seeking drivers in this setting may need the engagement of both quantitative and qualitative approaches as complementary approaches in future studies.

## LABORATORY PLATFORM

The collaboration between the laboratory scientists’ and epidemiologists’ expertise complement each other, and their combined efforts can yield public health data that are much stronger compared to the 2 disciplines working independently [34]. Thus, the KEMRI microbiology laboratory was established at the CGHR (Kisian campus) in 1998. The laboratory has rapidly expanded and formed 2 branches within the KEMRI-CGHR that includes the microbiology laboratory being managed purely by KEMRI while the other laboratory is managed by KEMRI and other partners. These laboratories are located at CGHR (Kisian campus), which is an approximately 40 km (1-hour drive) to the Siaya County HDSS area in western Kenya. The campus also hosts similar state-of-the-art laboratories that support various programs including malaria, human immunodeficiency virus, tuberculosis, respiratory, and neglected tropical diseases research activities.

To maintain the high-quality standards required in these studies, the laboratory has assembled a team of personnel with relevant qualifications and experience considering sensitivity to gender equity and room for mentorship of young scientists. Since quality assurance is paramount to our research platform, we have a quality assurance officer who ensures that all the laboratory staff abide by local and international quality and safety regulations [35, 36]. Moreover, all of our laboratory staff are trained in microbiology and molecular biology protocols before and during implementation of our studies. The knowledge gained in training and experience in medical laboratory techniques enables the laboratory team to support the field teams by occasionally visiting the field and training field staff and clinicians on specimen collection and transport guidelines in addition to providing relevant supplies. Being that KEMRI strongly advocates for capacity building and mentorship, the laboratory has committed to this course and is currently mentoring 3 BSc degree holders as junior scientists as they pursue their MSc courses, as well as several interns and undergraduate students on practical attachments.

To support quality research, the laboratory embraces quality assurance and Good Clinical Laboratory practices. The laboratory is well equipped with relevant microbiology and molecular test instruments, which undergo regular maintenance and quality checks with proper documentation and maintenance records. Therefore, the laboratory has the capacity to receive various biological specimens and performs microscopic examination, isolation (culture), identification, serotyping, and susceptibility testing of obligate and facultative anaerobes and microaerophilic microorganisms. The staff are also well trained and have practical experience in processing sample shipments abroad as per individual study protocols. Other capabilities include enzyme-linked immunoassays, conventional and real-time PCR, and TAC. Computers are available in the laboratory for data entry by the laboratory personnel. To ensure high quality of data, and without compromising the turnaround time, the results are reviewed at 2 levels before entry into the database or release to the field as feedback to the patients.

## TRAINING AND CAPACITY BUILDING

In line with EFGH coordination leadership goals, the EFGH Kenya leadership champions inclusivity and growth of junior investigators in the site and aims to uphold gender equity. Toward this goal, 10 different Kenyans were sponsored by the EFGH Consortium leadership in completing 3 online courses including Introduction to Epidemiology for Global Health, Fundamentals of Global Health Research, and Leadership and Management in Health from the University of Washington. Skills and experiences from the above trainings have equipped site team members to effectively undertake their roles and accomplish the objectives of the study. The distribution of mentorship and career development for young scientists in the Kenyan site is shown in Table 3. Two junior investigators were given the chance to attend the annual investigators meeting in Seattle, Washington, to take part in the project investigators meeting held in November-December 2022. Furthermore, 4 junior investigators have been included in the current EFGH protocol supplement papers to build their capacity in scientific writing. Our site also had 2 applications for the inaugural EFGH Rising Star Award, of which 1 was funded by the Consortium. One female junior investigator was also nominated to spearhead the EFGH inflammatory biomarker substudy under the mentorship of senior investigators. Two junior investigators with skills and experience in anthropometry were nominated by the University of Washington coordination team for EFGH to lead the development of anthropometric standard operating procedures and train the training-of-trainers from all the EFGH sites. Moreover, a female and a male junior investigator have been the recipients of the EFGH-supported manuscript writing certificate program award in 2023. While 37% of staff is comprised of females, we strive to increase gender diversity. Through EFGH, we have established a long-lasting research collaboration with several institutions including, but not limited to, the Center for Vaccine Development and Global Health, University of Maryland School of Medicine; Division of Infectious Diseases and International Health, Department of Medicine, University of Virginia; Department of Epidemiology, Rollins School of Public Health, Emory University, Atlanta; and Global Center for Integrated Health of Women, Adolescents and Children, Department of Global Health, University of Washington.

**Table 3.** Distribution of Mentorship and Career Development for Young Scientists in the Kenyan Site for Enterics for Global Health–*Shigella* Study Platform, May 2023

Distribution	No. of Senior Scientists and Qualification	Current No. of Scientists Pursuing PhD	No. of Young Career Scientists Pursuing Master’s Degree	Annual Recruitment Rate for Interns Pursuing Practical Skills
Department				
Epidemiology/data	1	2	5	8
Laboratory	2	0	3	4
Sex				
Male	3	2	3	8
Female	0	0	5	4

## CONCLUSIONS

Through GEMS, VIDA, and other collaborative projects, we have demonstrated use of a collaborative approach to engage synergy in assembling a research team with the essential skills and scientific expertise necessary to conduct epidemiology and clinical trials related to pediatric enteric and other infectious diseases as described elsewhere [1, 37–40]. A description of our site experience and infrastructure is outlined in Table 4, demonstrating our ability to contribute data needed to successfully implement the planned EFGH-*Shigella* surveillance with intention to advise and prepare potential sites for a vaccine trial.

**Table 4.** Summary of Experience and Infrastructure of the Kenya Medical Research Institute–Based Diarrhea Disease Surveillance Platform, Western Kenya, 2007–2022

Study Description	Multicountry or Multicenter Study	Targeted Population	Enrollment, No.	Follow-up Period	Retention Rate	Data Collection Method
Global Enteric Multicenter Study (GEMS) [1]	Yes	Children aged 0–59 mo with MSD and control	3359	60–90 d	97%	Paper-based with optical character recognition software and use of Safe File Transmission Program Server
Vaccine Impact on Diarrhea Assessment (VIDA) [18]	Yes	Children aged 0–59 mo with MSD and control	3649	60–90 d	97%	Real-time EDC–Advantage
Phase 3 rotavirus trial [38]	Yes	Infants aged 4–12 wk	1308	24 wk	98%	Visual Basic for .NET applications electronic system
RCT of ceramic water filter [39]	No	Infants aged 4–10 mo in 240 households	240	25 wk	97%	Paper-based with optical character recognition software
Rotavirus Immunization Program Evaluation in Kenya (RIPEK) [40]	Yes	Children aged 0–59 mo with acute gastroenteritis	…	…	97%	VB.Net electronic system
Enterics for Global Health (EFGH)–*Shigella* Study	Yes	Children aged 6–35 mo with medically attended diarrhea	…	90 d	…	REDCap and SurveyCTO

Abbreviations: EDC, electronic data capture; MSD, moderate to severe diarrhea; RCT, randomized controlled trial; REDCap, Research Electronic Data Capture.

## Notes

***Acknowledgments.*** We appreciate the contributions and efforts of KEMRI-CGHR staff in all domains of the study (diarrhea case surveillance, population enumeration, and laboratory) involved in the data collection, data management, and laboratory testing of samples. We also wish to thank the study participants at each of the recruitment centers, the KEMRI leadership, and Ministry of Health and county health staff for supporting this study as well as previous studies. Additionally, we appreciate the technical support from the EFGH Consortium and University of Maryland, Baltimore. This manuscript is published with permission of the KEMRI Director.

***Financial support*.** This research was supported by the Bill & Melinda Gates Foundation (grant numbers INV-016650, INV-031791, INV-028721, INV-041730).

***Supplement sponsorship.*** This article appears as part of the supplement “Enterics for Global Health (EFGH) *Shigella* Surveillance Study-Rationale and Methods,” sponsored by the Bill & Melinda Gates Foundation.
